# Generation of a Comprehensive Transcriptome Atlas and Transcriptome Dynamics in Medicinal Cannabis

**DOI:** 10.1038/s41598-019-53023-6

**Published:** 2019-11-12

**Authors:** Shivraj Braich, Rebecca C. Baillie, Larry S. Jewell, German C. Spangenberg, Noel O. I. Cogan

**Affiliations:** 1 Agriculture Victoria Research, AgriBio, Centre for AgriBioscience, Bundoora, Victoria 3083 Australia; 2 School of Applied Systems Biology, La Trobe University, Bundoora, Victoria 3086 Australia

**Keywords:** Plant sciences, Plant development, Plant genetics, Plant molecular biology, Plant reproduction, Computational biology and bioinformatics, Agricultural genetics, Gene expression, Plant genetics, Sequencing, Next-generation sequencing, RNA sequencing, Sequence annotation, Transcriptomics

## Abstract

Cannabinoids are the main medicinal compounds of interest in the plant *Cannabis sativa*, that are primarily synthesised in the glandular trichomes; found on female floral buds. The content, composition and yield of secondary metabolites (cannabinoids and terpenoids) is influenced by the plant’s genetics and environment. Some initial gene expression experiments have been performed from strains of this plant species that contrasted in cannabinoid production, however the present knowledge about detailed trichome transcriptomics in this species is limited. An extensive transcriptome atlas was generated by RNA sequencing using root, shoot, flower and trichome tissues from a female plant strain (Cannbio-2) and was enhanced with the addition of vegetative and reproductive tissues from a male cannabis plant. Differential gene expression analysis identified genes preferentially expressed in different tissues. Detailed trichomics was performed from extractions specifically from glandular trichomes as well as female floral tissues at varying developmental stages, to identify stage-specific differentially expressed genes. Candidate genes involved in terpene and cannabinoid synthesis were identified and the majority were found to have an abundant expression in trichomes. The comprehensive transcriptome is a significant resource in cannabis for further research of functional genomics to improve the yield of specialised metabolites with high pharmacological value.

## Introduction

The plant genus *Cannabis* within the Rosales clade of eudicot angiosperms is a member of the plant family Cannabaceae. Cannabis is a diploid species (2n = 20) consisting of a karyotype of a pair of sex chromosomes (X and Y) and nine autosomes^1^ with a dioecious system, although monoecious forms can exist. Cannabis has recently gained a revival of interest, due to the increasing legalisation for medicinal uses across the globe. Production of phytocannabinoids or more simply plant cannabinoids, is the most prominent feature of this plant that contributes to its’ unique pharmacological properties. Cannabinol (CBN) was the first pure isolated compound from the exuded resin of Indian hemp^2^ which was followed by the discovery of cannabidiol (CBD)^3^. In 1964, the active compound delta-9-tetrahydrocannabinol (Δ^9^-THC, or simply THC) was isolated^4^. The principal pharmacological effects of CBD (non-psychoactive cannabinoid) include muscle relaxant, anticonvulsant, neuroprotective, antioxidant, anxiolytic and antipsychotic activity and has been responsible in decreasing the psychoactive and anxiogenic effects of THC^5^. THC is the primary recreational drug, however it is known to have pharmacological characteristics to be utilised for analgesia, appetite stimulation, antiemesis and muscle relaxation^5^. *Cannabis sativa* L. has been reported to produce a total of 565 constituents including at least 120 cannabinoids^6,7^.

Trichomes are generally defined as unicellular or multicellular structures, which develops from epidermal cells. Based on the secretion ability and morphology, trichomes are categorised into glandular and non-glandular types, and cannabis exhibits both these types of trichomes^8^. Secretory cells inside glandular trichomes are reported to be exclusively specialised structures that synthesise high amounts of secondary metabolites^9^. Trichomes are present on most aerial parts of the plant^8^ with highest density on the floral buds of the female plant. Other parts of the plants such as seeds, roots and pollen also produce phytocannabinoids but in low quantity^10–12^. Although the cannabis plant is primarily known for its production of cannabinoids (especially THC and CBD), the resin is also known to contain a variety of terpenoids, phenylpropanoids/polyketides, acyl sugars and fatty acid derivatives^9,13^. Terpenes are primarily responsible for providing aroma and characteristic flavours and are also known to have pharmacological effects including antibacterial, anti-inflammatory, analgesic, anti-anxiety, anxiolytic and sedative effects^14^. The yield of cannabinoids and terpenes is influenced by the genetic constitution of the plant and its environment^15–17^. In this context, genes are responsible for determining the plant’s chemotype, density of trichomes, size of resin heads and the gender of the plant with some influence of the environment. It is in female flowers that the higher concentration of glandular trichomes is found, making these organs the main cannabinoid producers^18^.

Unlike other economically important crops, cannabis has only recently started to gain genomic resources, in the form of whole genome sequences^19–21^, but is limited in functionally associated gene expression data. A study in this species^18^ assembled transcriptomes from the Purple Kush (PK) strain using roots, stems, shoots and flowers (pre-flowers, early-stage and mid-stage flowers) and from the hemp cultivar ‘Finola’ using the mid-stage flowers. PK and Finola transcriptome assemblies were used to analyse variation in expression of the cannabinoid and precursor pathway genes in PK and Finola cultivars. The pathway that generates CBD and THC has been extensively studied^18,22,23^, however minor cannabinoids, terpenes and flavonoids have not been studied in depth in terms of their biosynthesis. Identification and subsequent targeted modification of genes responsible to produce these metabolites of interest requires detailed genomic and chemotypic information.

The current study reports on the generation of a comprehensive transcriptome assembly, Cannbio assembly using RNA-Seq from a female cannabis plant strain, Cannbio-2 which was enhanced by leaf and reproductive flower data from a male cannabis plant strain, Cannbio-male. Specific key targets of female floral buds and trichome tissues that were sampled at various developmental stages, were characterised in this study. Differential gene expression profiles in all plant tissues and across female flower developmental stages were analysed. Furthermore, the expression level of genes involved in terpenoid and cannabinoid synthesis identified from ‘Finola’ resin^24^ was compared in various tissue types and across female flower developmental stages in trichomes. The results from this study are significant for prediction of cannabinoids and terpenes composition and for selection based on phytochemical diversity which can be further studied in future research.

## Results

### RNA-Seq and de novo transcriptome assembly

A total of seventy-one RNA-Seq libraries were sequenced aiming to obtain a minimum of 30 million reads from each sample. The transcriptome assembly was generated from a total of 6,946,497,370 sequence reads. A complete list of 71 samples and associated details used in the *de novo* transcriptome assembly is provided in Supplementary Table S1.

The high-quality trimmed reads were initially assembled using the SOAPdenovo-TRANS assembler. An empirically optimised k-mer value of 73 was used for the assembly. The statistics of the sequencing data filtering and outputs are summarised in Table 1, with the initial assembly resulting in 500,485 contigs and scaffolds with a mean size of 487 bp. Following the initial assembly, a total of 221,849 contigs were removed as they had length less than 240 bp (considerably shorter than a pair of sequence reads) and were considered likely to be spurious. A further total of 94,670 contigs were also removed, as they had less than 10 sequence reads associated with the initial assembly and their length ranged between 240–500 bp. These filtering steps removed a large number of transcripts and resulted in a total of 183,966 contigs and scaffolds remaining.

**Table 1:** Sequencing outputs and transcriptome assembly statistics of the primary, secondary and filtered Cannbio assembly.

Assembly	Statistics
***Primary Assembly: SOAPdenovo-Trans***	
Total number of transcripts	500,485
Total base pairs (without N)	241,253,446 bp
N50 length	954 bp
***Secondary Assembly: CAP3***	
Number of transcripts	143,671
Total base pairs	104,880,973 bp
N50	1071 bp
***Final Assembly: Filtered***	
Number of transcripts	64,727
Total base pairs	57,300,518 bp
N50	1846 bp

The initially assembled scaffolds (57,268) that were identified as fork, bubble and complex loci in nature from the SOAPdenovo-TRANS assembly were individually assembled using CAP3. The CAP3 assembler resolved 24,840 scaffolds relating to 7,143 loci (each representing a single sequence in the transcriptome assembly). The majority of scaffolds that were not resolved by the CAP3 assembly step, were complex loci (78.9%). The unresolved scaffolds (32,428) were analysed, and a single longest transcript for each locus from these scaffolds was retained in the assembly, this added another 9,830 transcripts to the assembly. The secondary enhanced Cannbio assembly (Table 1) resulted in 143,671 contigs and scaffolds with N50 of 1071 bp and N90 of 287 bp with the largest transcript length of 167,637 bp.

### Classification and annotation of the transcriptome assembly

**Figure 1:**
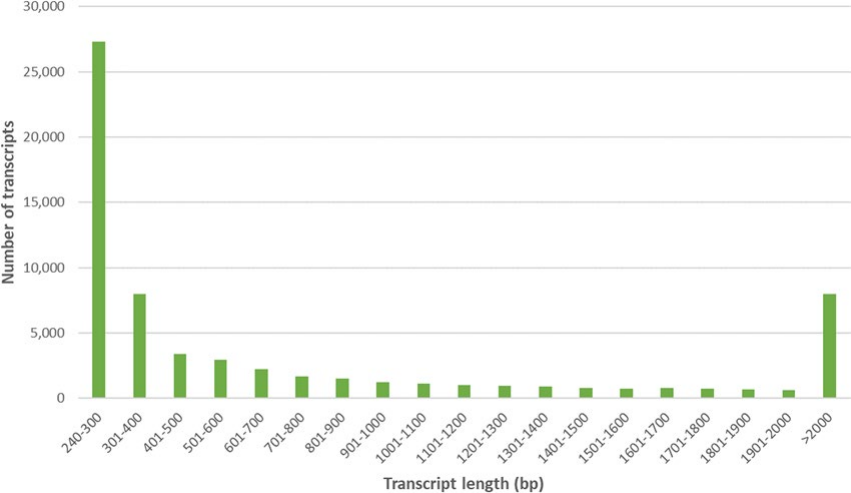
Distribution of the contig and scaffold length from Cannbio transcriptome assembly.

The secondary assembly was used as the query file for a BLASTX search against UniRef100 database and identified 82,610 transcripts corresponding to 53,652 unique UniRef100 identifiers. Contigs and scaffolds that were not annotated by UniRef100 BLASTX search were removed from the transcriptome assembly. Based on the obtained annotation of the UniRef100 protein, a total of 19,440 transcripts exhibited the highest matches to sequences of non-plant derived sources. A small percentage of these non-plant identified transcripts (0.08%) showed high-value matches of moderate similarity to the published cannabis transcriptome assemblies of PK and Finola^18^ and were therefore retained in the assembly, all other non-plant identified sequences were removed from the assembly. Out of the 61,061 unannotated sequences, 36,392 transcripts displayed similarity matches to either or both PK and Finola transcriptome assemblies (Supplementary Table S2) but were not included for further analysis as they failed to return a match to a known protein. The final filtered Cannbio transcriptome assembly comprised of 64,727 contigs and scaffolds (Table 1). The size distribution of the final Cannbio transcriptome assembly was determined (Fig. 1). The majority of the contigs and scaffolds ranged between 240–300 bp in length (42.2%), followed by those that were above the length of 2000 bp (12.3%) with the largest transcript length of 107,602 bp and N50 of 1,847 bp.

**Figure 2:**
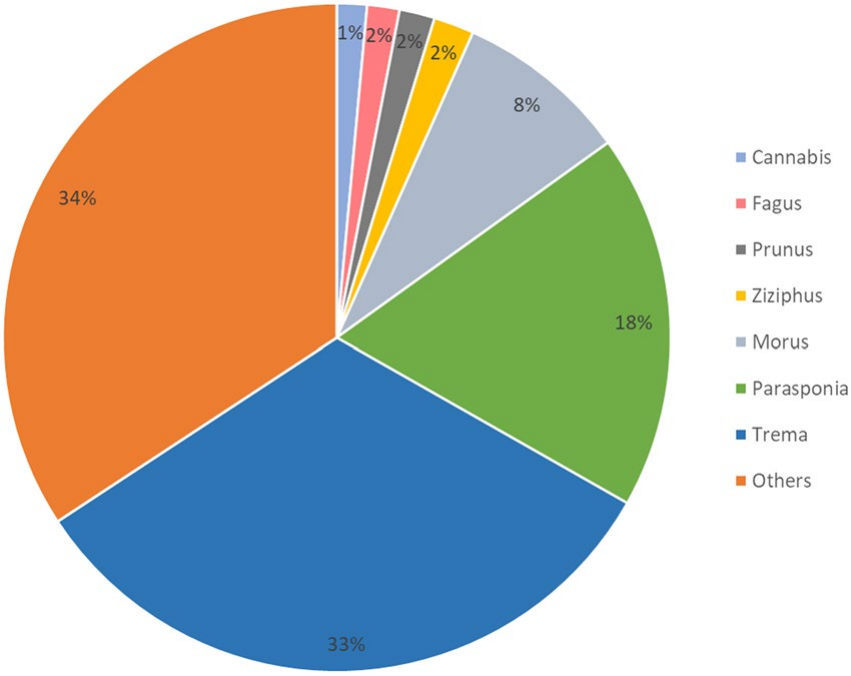
Genus distribution of the Cannbio characterised transcripts similarity based on UniRef100 annotation.

The BLASTX analysis to the UniRef100 database also revealed the distribution of similarity of the assembled transcripts to other plant species (Supplementary Table S3). Figure 2 represents the genus wide similarity distribution of the transcripts from the current study that was obtained from the taxonomy of the corresponding similar protein. A total of 21,012 transcripts displayed the highest similarity to *Trema orientalis*, followed by *Parasponia andersonii* (11,721) and *Morus notabilis* (5,363).

Comparison of the final Cannbio transcriptome assembly to the previously published cannabis transcriptome and CDS datasets revealed that the current assembly captured 89% of the transcripts of PK^18^, 93.7% transcripts of Finola^18^ and 78.7% of the coding sequences (CDS) of the CBDRx assembly^19^ (Supplementary Table S4). A total of 48,893 of the Cannbio assembly transcripts were present in all three datasets, while 2,726 of the contigs and scaffolds were found to be exclusive to the Cannbio assembly and have not been previously characterised in this species’ datasets.

**Figure 3:**
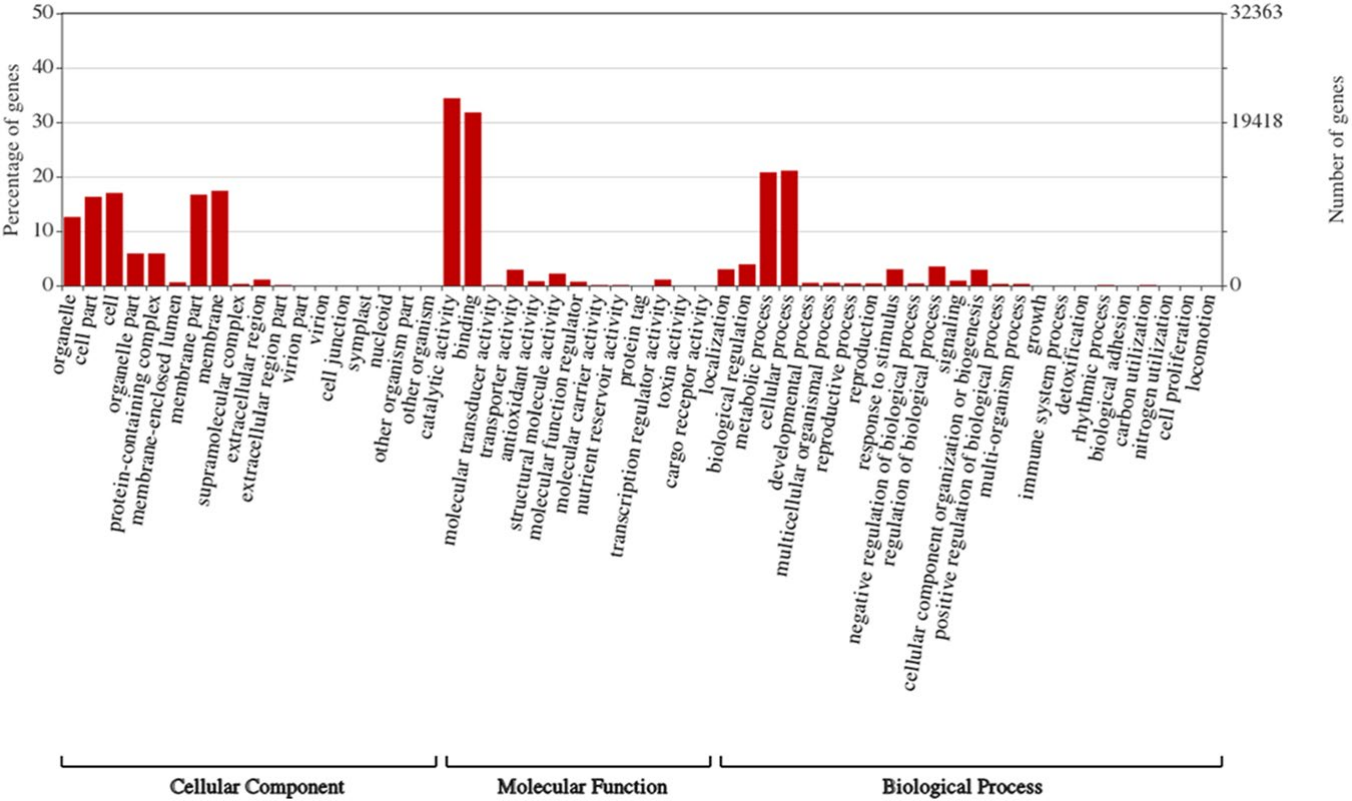
Gene ontology (GO) terms assignment for the Cannbio transcriptome. Results are summarised into three main GO categories of cellular component, molecular function and biological process. The left y-axis represents the percentage of specific category of genes present in each main category whereas, the right y-axis indicates the gene number in the same category.

Gene function categories of the contigs and scaffolds generated from the current transcriptome assembly were obtained by assigning GO terms based on the sequence similarity to UniRef100 database. A total of 41,457 transcripts from the assembly were assigned at least one GO term (Fig. 3). GO assignment was recorded to be the highest for molecular function (47.3%), followed by cellular component (27.8%) and biological process (25%). Amongst the annotated sequences, molecular function categories included catalytic activity (22,272), binding (20,593), transporter activity (1,881), structural molecule activity (1,406) and other categories (1,851). Cellular component categories included membrane (11,250), cell (11,019), membrane part (10,789), cell part (10,578), organelle (8,176) and other categories (9,082). In addition, biological process categories were comprised of cellular process (13,640), metabolic process (13,447), biological regulation (2,546), regulation of biological process (2,288), localization (1,926), response to stimulus (1,911), cellular component organization or biogenesis (1,884) and other categories (2,545).

### Differential gene expression analysis

**Figure 4:**
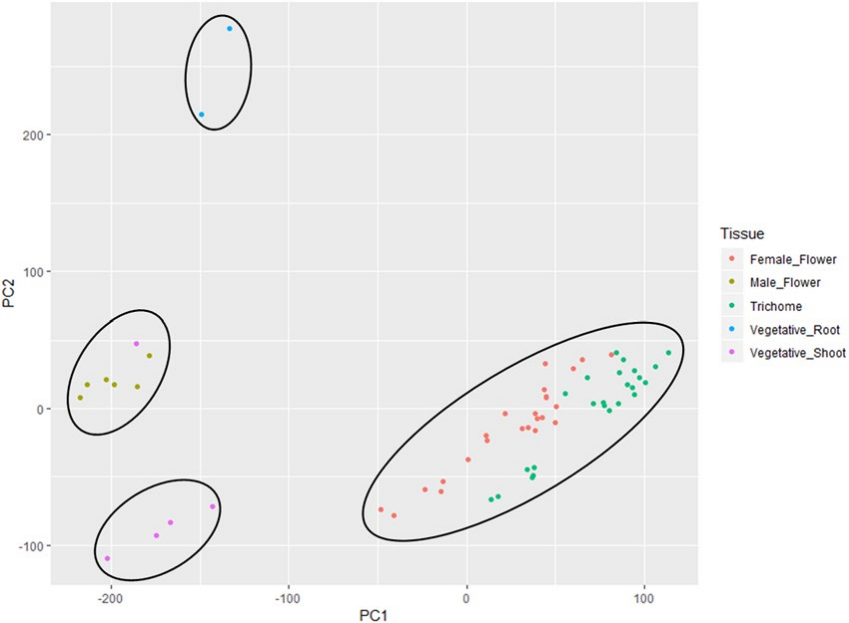
PCA plot of various tissue types included in the Cannbio assembly based on their normalised read count data.

Following normalisation of read counts, similarity between samples of various tissue types was assessed by plotting a principal component analysis (PCA) graph from the normalised count data (Fig. 4). Normalised data from read counts obtained from each tissue type formed four distinct clusters of root tissues, shoot tissues (with one outlier), female floral and male floral tissues.

**Figure 5:**
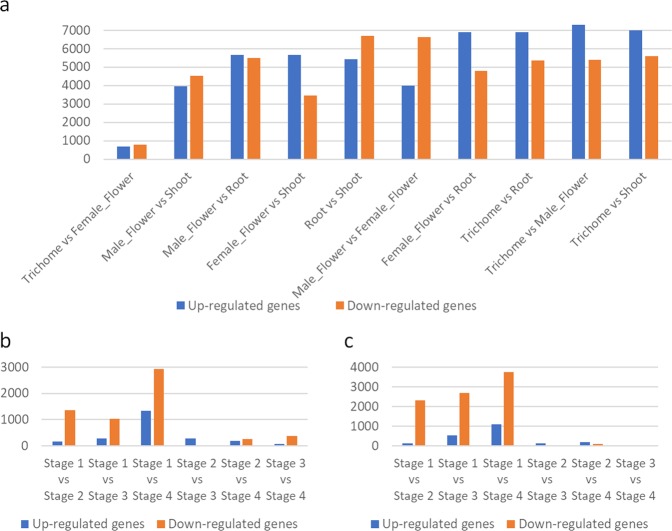
Number of differentially expressed genes amongst various tissue type (**a**) and amongst various developmental stages of flower development in the tissues of female flower (**b**) and trichomes (**c**).

Comparisons of gene expression were made between the distinct tissue types to identify differentially expressed genes as represented in Fig. 5a. Comparisons between trichome and female flower tissue (with retained trichomes) revealed the least divergence in gene expression with only 1,479 differentially expressed genes (46.4% up-regulated and 53.6% down-regulated genes) in trichomes when compared to female flowers with log2FoldChange ranging from −14.9 to 6.2. Female floral tissues, especially the trichomes were found to be the most distinct group due to the maximum divergence from all other tissue types. A total of 12,669, 12,598 and 12,277 differentially expressed genes were found in trichomes as compared to male flower, vegetative shoot and root tissues respectively. Glycoside hydrolase, naringenin-chalcone synthase, lipoxygenase and sieve element occlusion genes were the most frequently found gene nomenclature that was up-regulated in trichomes. Additionally, common cannabis allergens especially, Betv1 and non-specific lipid transfer proteins (ns-LTPs) and genes identified as Light Oxygen Voltage (LOV) domain containing proteins were also found to be significantly up-regulated in female flowers and trichome tissues. Comparisons between female and male reproductive floral tissues identified genes that were most commonly up-regulated genes in male flowers annotated as leucine-rich repeat (LRR) and F-box domain containing proteins, pseudo-autosomal region (PAR) and endonucleases. All the significant differentially expressed genes with their annotations based on UniRef100 database similarity results and log2FoldChange value across different comparisons between tissue types are detailed in Supplementary Table S5.

The number of genes that were identified to be differentially expressed across various developmental stages in female flowers and trichome tissues were also analysed and are represented in Fig. 5b,c. It was found that developmental Stage 1 had the most divergent dataset when compared to all other stages in terms of gene expression. A notable increase in the number of up-regulated genes was observed at Stage 4 when compared to Stage 3, Stage 2 and Stage 1 in both the female flowers and trichomes. For instance, Stage 1 (immature floral bud) when compared to Stage 4 (mature floral bud) had 4,274 (31.2% up-regulated and 68.8% down-regulated genes) and 4,854 (22.6% up-regulated and 77.4% down-regulated genes) differentially expressed genes in female flowers and trichomes respectively. The genes that were found to be frequently up-regulated in Stage 1 when compared to Stage 4 in female flowers and trichomes had similar gene annotations; for example, sieve element occlusion, lipase, cytochrome P450 and fatty acid hydroxylase. In female flowers, the gene expression was observed to change the least in Stage 2 when compared to Stage 3 (296 genes), followed by either Stages 2 and 3 as compared to Stage 4. Whereas in trichomes, the least expression change was found in Stage 3 when compared to Stage 4 (37 genes), followed by Stage 2 as compared to Stages 3 and 4. All the significant differentially expressed genes identified based on comparisons made across the female reproductive developmental stages with their UniRef100 annotations and log2FoldChange are detailed in Supplementary Tables S6a,b for trichomes and female flowers respectively.

The number of differentially expressed genes between Stages 1 when compared to Stage 4 were found to be maximum and these genes were further categorised functionally based on their GO term (Supplementary Fig. S1). The majority of the enriched genes in each comparison were attributed to a functional category, in which the most frequent categories were “catalytic activity” and “binding”; followed by biological and cellular categories. The GO category for biological process revealed that the number of enriched genes in the two types of “metabolic process” and “cellular process” was the largest. The most prevalent GO categories for cellular component included “membrane” and “membrane part”.

Furthermore, quantitative reverse transcription polymerase chain reaction (qRT-PCR) analysis revealed that all the genes exhibited similar expression patterns in qRT-PCR as observed in the RNA-Seq data (Supplementary Fig. S2). A high proportion of the transcripts (17 out of 20) had a correlation coefficient of ≥0.96. The remaining three transcripts displayed slight discordant outcome with Pearson’s correlation coefficient ranging between 0.93 and 0.94.

### Expression analysis of genes involved in terpene and cannabinoid biosynthesis

BLASTN searches against the genes involved in terpene synthesis identified 124 transcripts from the plastidial methylerythritol phosphate (MEP) pathway, 69 transcripts from the cytosolic mevalonate (MEV) pathway and 24 transcripts as prenyltransferases from the current assembly (Supplementary Table S7). A total of 136 transcripts were identified to represent the cannabis terpene synthases (CsTPS) out of which CsTPS1FN was found to be the most abundant in the current assembly followed by CsTPS8FN, CsTPS2FN and CsTPS3FN (Supplementary Table S7). In addition, a total of 30 transcripts were identified as tetrahydrocannabinolic acid synthase (THCAS) or cannabidiolic acid synthase-like 1 (CBDAS- like 1) or cannabidiolic acid synthase (CBDAS) based on the annotation of similarity results to UniRef100 database.

**Figure 6:**
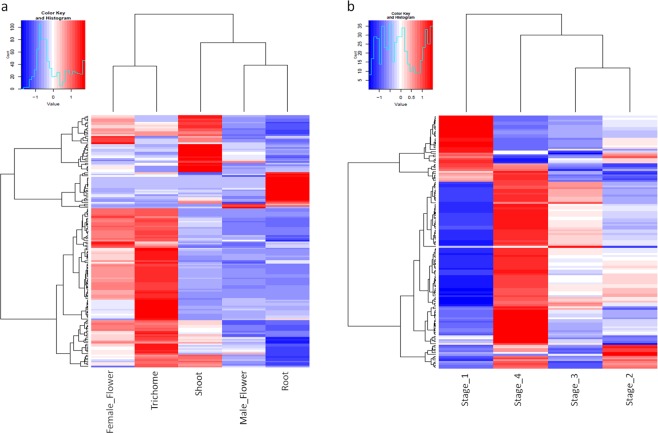
Heatmaps of the differently expressed transcripts of interest representing hierarchical clustering across the various tissue types (**a**) and the developmental stages in trichomes (**b**). The normalised log transformed counts are indicated by the colour key. Red indicates high expression, white represents intermediate expression and blue is indicative of low expression in the heatmaps.

The relative level of expression for the identified candidate transcripts of interest in each tissue type is represented in Fig. 6a. It was found that most of these genes involved in terpene synthesis had high expression in the female floral tissues, especially trichomes with some exceptions. For instance, root tissues were found to have higher expression of cannabis 1-deoxy-D-xylulose 6-phosphate (DOXP) synthase (CsDXS)2 involved in MEP pathway; cannabis HMG-CoA reductase (CsHMGR)1, cannabis mevalonate kinase (CsMK), cannabis mevalonate-5-phosphate decarboxylase (CsMPDC), cannabis phospho-mevalonate kinase (CsPMK) involved in MEV pathway and prenyltransferase cannabis farnesyl diphosphate (FPP) synthase (CsFPPS)1. Whereas, trichomes exhibited higher expression of CsDXS1, CsHMGR2 and CsFPPS2 when compared to other samples. Additionally, the majority of terpene synthase genes were highly expressed in the female flowers with some outliers. The relative expression analysis revealed CsTPS5FN, CsTPS9FN and CsTPS12PK gene models were typically expressed at heightened levels in the vegetative root and/or shoot tissues compared to floral tissues. Genes representing CBDAS and THCAS were found to have higher expression in the trichomes; whilst, CBDAS-like 1 was found to have highest expression in the male flower.

Trichomes were found to be significantly enriched in terms of expression for the genes of interest therefore, the relative expression level of these genes was analysed in trichomes across the developmental stages (Fig. 6b). The analysis revealed that the majority of the genes involved in the MEP pathway had high expression levels at Stage 4 of flowering; whereas, the majority of the MEV pathway genes have relatively higher expression during the earlier stages of flower development (Stage 2 and Stage 1). Prenyltransferases (except CsFPPS1), the majority of terpene synthases (except CsTPS4FN, CsTPS5FN that had variable expression and CsTPS13PK had high expression in Stage 1), CBDAS and THCAS genes also had relatively higher expression in the latter stages of female flower development (mature floral buds) compared to immature floral buds.

## Discussion

Flower development is a key feature for the majority of plants, defining the reproductive phase of the plant and is of even more significance in cannabis, due to cannabinoid production. Understanding gene expression regulation for plant organogenesis and associated molecular mechanisms will provide insight to cannabis flowers and secondary metabolites production. A few genome-wide studies have been performed to understand the transcriptional changes during reproductive development in plants, exemplified by rice^25^, chickpea^26^, sugar apple^27^, *Populus*^28^, China rose^29^. There are two available transcriptomes of *C. sativa* which were generated from roots, shoots and flowers from different stages of a high-THC female strain PK, and from female flowers of hemp cultivar Finola^18^. Flowering stages from PK included pre-flowers (shoot tips without visible stigmas), early-stage (flowers with visible stigma) and mid-stage (flowers with non-withered stigmas and visible trichomes) and from Finola only the mid-stage flowers were harvested. A previous transcriptome study^18^ focussed on variation in gene expression in the cannabinoid pathway for THC and CBD production between the two cultivars. Furthermore, previously published transcriptome studies in this species^18,19^ lacked extensive sampling and sequencing depth, to make statistically relevant and robust datasets. However, a comprehensive analysis in the expression of genes based on tissue type and developmental stage during female flower development, with the additional dissection of trichomes tissues was lacking for the species. Moreover, the male cannabis plant tissue was not sampled previously. The current study aimed to describe a global view of gene expression dynamics during female flower development and tissue-specific expression. The comprehensive statistically relevant transcriptome dataset has provided insights into the transcriptome dynamics in cannabis.

The study reports the generation of a *de novo* transcriptome assembly using a range of source tissues including vegetative plant’s shoot and root tissues, different developmental stages of female flower development and the male flower of *Cannabis sativa*. Deep sequencing of the RNA-Seq libraries was done to enable representation of lowly expressed tissue-specific transcripts. The number of raw reads generated using RNA sequencing (c. 7 billion) represents a significant advance compared to those previously published in this species^18,30,31^.

Not all contigs and scaffolds from the generated transcriptome assembly were annotated based on similarity search to known protein of UniRef100 database which could represent the presence of pseudogenes, or repetitive elements, or genes with disrupted function which were discarded from the assembly. Moreover, the subset of transcripts was found to be from non-plant origin and this level of contamination was found to be comparable in other plant datasets^32^ which could indicate the presence of microbial communities in the rhizosphere, and in planta. Cannbio’s transcripts had the highest number of similarity matches to *Trema orientalis* and *Parasponia andersonii*, which is consistent with known phylogenetic relations, as all belong to the Cannabaceae^33^. Moreover, molecular results have suggested that *Parasponia* is found nested within *Trema*^34^ and more recently the *Parasponia*-*Trema* clade were found to be taxonomic sisters (strong bootstrap support = 100) by plastome sequences^35^.

A total of 95.8% of Cannbio’s transcripts displayed similarity to previously published transcriptome studies^18,19^, while the remaining 4.2% were found to be mostly annotated as uncharacterised proteins in other plant species. The uncharacterised transcripts may represent rarely expressed genes that may be strain-specific or sequencing artefacts. Furthermore, the final assembly had an N50 of 1,847 bp which was higher than the previously published cannabis transcriptomes of PK (1,804 bp)^18^, Finola (1,193 bp)^18^ and the CDS of the CBDRx genome assembly (1,482 bp)^19^. A higher N50 is likely due to the presence of untranslated regions (UTRs) in this study as compared to previous datasets. In terms of functional characterisation of the transcripts, 64% of Cannbio’ transcripts were assigned at least one GO term, which is comparable with other published plant transcriptome studies^32,36,37^.

The PCA analysis generated from the normalised read count of the tissues analysed in the current study revealed that the tissues fell into four major clusters based on the transcriptional activity. The tissues that were included in these major groups represented similar plant structures. Trichomes displayed the least divergence from female flowers which is likely due to the impracticality of removing the trichomes from female flowers in this transcriptome study. Specific genes were identified that were preferentially tissue expressed and differentially expressed from immature to mature buds in female flowers. Many of the stage-specific genes involved in the development of female flowers identified in this study can further help to functionally characterise genes that can be of potential interest for future studies in cannabis. For instance, sieve element occlusion genes were significantly upregulated in female flowering tissues and these genes are known to have a role in blocking damaged sieve elements after injury to prevent loss of nutrients (sap) in flowering plants^38^. In opium poppy, sieve elements produce and accumulate benzylisoquinoline alkaloids which are a diverse group of biologically active and specialised metabolites involved in morphine biosynthesis^39^. Moreover, some of the known cannabis allergens including Bet v 1, the major cannabis pollen allergen and ns-LTPs^40^ were also upregulated in female flowering tissues^40,41^. The transcripts that were identified to represent these allergens in this study can be useful in characterisation of cannabis allergens and in the development of hypoallergic cannabis plants.

Changes in the gene expression levels during every developmental stage of female flowers and trichomes (especially Stage 1 which is the immature bud to all other stages), indicated that the flower development may be controlled by complex transcriptional regulation. Differential expression between Stage 1 and Stage 4 revealed an enrichment in the “catalytic activity” and “binding” within the GO molecular function category. The GO molecular function categorisation was found to be consistent with a specialized role in defence and specifically in chemical defence as the process is heavily dependent on catalytic activity essential for the production of flavonoids, phenolics, glucosinolates, terpenoids, and alkaloids^42^. Furthermore, the GO biological process category indicated enrichment in “metabolic process” and “cellular process”. The GO category of cellular component revealed that the differentially expressed genes were most frequent for “cell”, “cell part”, “organelle”, and “membrane” during floral bud differentiation. Combining the changes observed in GO terms broadly, a clear picture of cellular turnover in metabolism and defence related compounds emerges that clearly involves a significant number of genes and their related proteins.

The correlation results obtained using qRT-PCR for the majority of the selected transcripts (85%) were found to be consistent with the expression patterns identified by RNA-Seq. Transcripts that displayed an inconsistent expression pattern between RNA-Seq and qRT-PCR data could be due to relevant primer pairs’ lack of specificity resulting in non-specific amplification, or detection of paralogous gene sequence’ expression profile. The validation results were found to be comparable with other published plant transcriptome studies^36,37^.

Expression profiles of the key aspect of cannabis, cannabinoid and terpenoid synthesis, were analysed across tissue types and developmental stages of female flowers. A previous study has characterised genes involved in terpene synthesis and found that *TPS* genes and MEP and MEV pathways’ gene transcripts were expressed in floral trichomes at a high level^24^. Expression profiles of these characterised genes in the current study was found to be consistent with the results previously reported^24^. In addition to this, vegetative root and shoot tissues were found to have high expression of certain terpene synthases (CsTPS5FN, CsTPS9FN and CsTPS12PK) when compared to female flowering tissues. The present study also highlights that terpene and cannabinoid gene expression varies based on the developmental stage. For instance, the majority of the terpene synthases were highly expressed in mature floral buds, expression of CsTPS13PK (encoding major product, (Z)-β-ocimene^24^) was found to be highest in immature floral buds when compared to mature buds. The variation in gene expression is likely to influence secondary metabolite production. Normalisation of developmental stage could be critical to standardise the harvest of female floral buds for resin production.

The current study is the first study in this species providing in-depth insights into genome-wide transcriptome dynamics across various tissue types and during female flower development (especially trichomics). A large set of candidate genes was identified as an outcome of this study, which apparently not only play a role in terpenoid and cannabinoids synthesis but also flower development stage-specific and tissue type differentially expressed genes. The detailed analysis of the tissue-preferential and development stage-specific genes provided will assist future studies in understanding the molecular mechanisms involved in the initiation and development of the floral buds. The results from the current study contributes as a significant resource that can be used in future cannabis plant selection or breeding, or to modify plants by genome-editing to generate preferred varieties.

## Conclusion

The study presents a comprehensive transcriptome assembly, generated using RNA-Seq technology. By extensive sampling of various vegetative and reproductive tissue types, a transcriptome atlas of gene expression has been created and annotated. A large set of genes has been identified that are differentially expressed in various tissue types and across developmental stages of female flowers. Candidate genes involved in terpene and cannabinoid synthesis were also identified and quantified for gene expression. The dataset generated in this study will be invaluable to annotate emerging whole genome assemblies and will also provide as a critical resource for a range of applications including functional genomics and breeding.

## Methods

Details of the experimental procedure followed in the study are summarised in Supplementary Fig. S3 (using R package, DiagrammeR).

### Plant material

**Figure 7:**
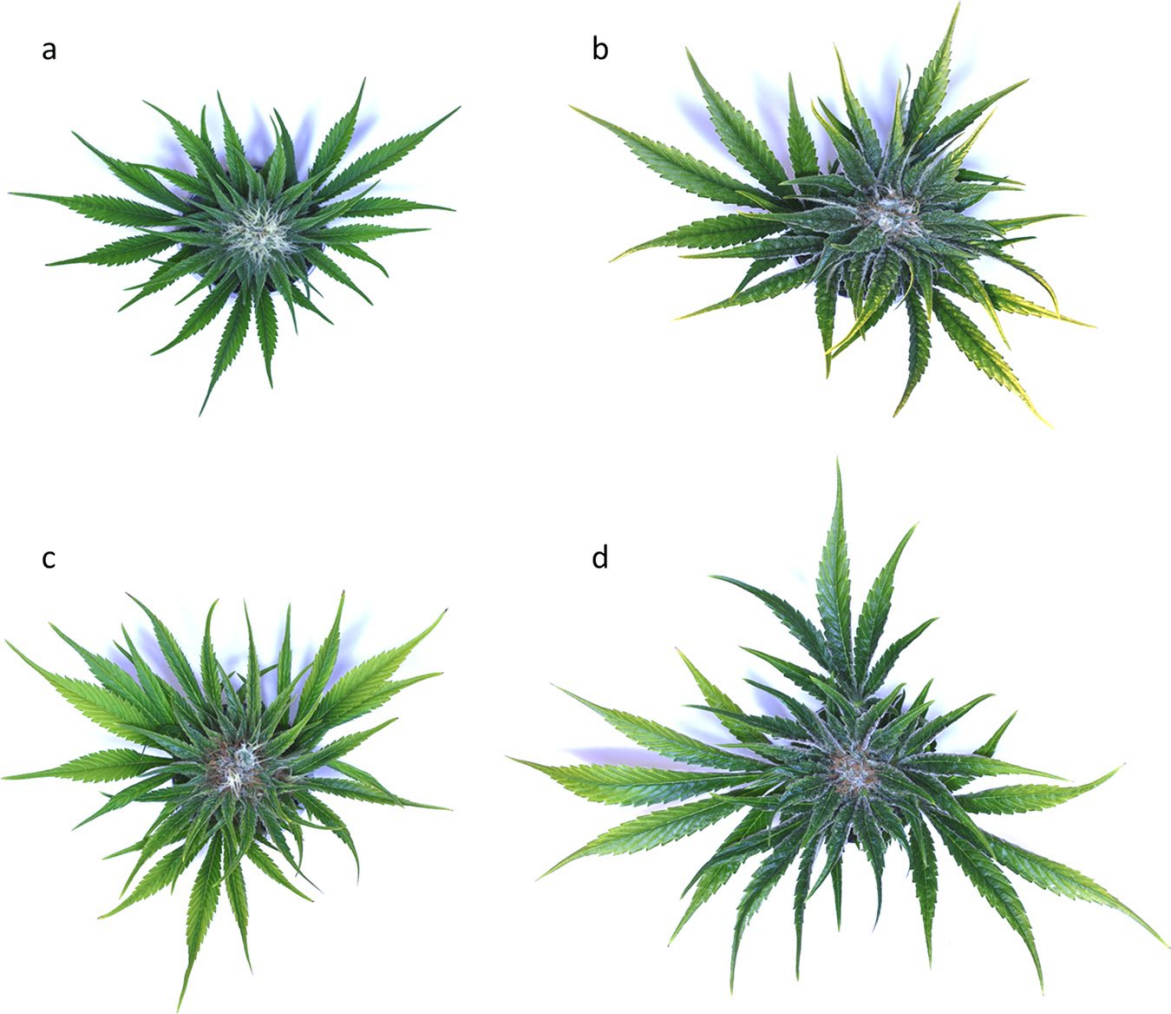
Floral buds of reproductive plant at (**a**) 35 days, (**b**) 42 days, (**c**) 49 days and (**d**) 56 days post-induction of flowering. Maturation of the flower can be seen in the trichome content of the sugar leaves as well as the colour change of the pistil from white (immature bud) to brown (mature bud).

Clonal copies of the female strain Cannbio-2 and male strain Cannbio-male were maintained under artificial conditions in controlled environment facilities. All the work undertaken was performed under Medicinal Cannabis Research Licence (RL011/18) and Permit (RL01118P6) issued by the Department of Health (DoH), Office of Drug Control (ODC) Australia. For transcriptome atlas development, plant tissues from multiple sources (Supplementary Table S1) were sampled including stem, root-tip, root-mid, leaf tissue at various developmental stages of the plant that ranged from a freshly planted cutting, vegetative plant to reproductive plant. To study the expression level of the cannabinoid biosynthesis pathway genes, floral bud tissues and trichomes were isolated from reproductive plants at four different timepoints, in six biological replicates. The four timepoints included tissues harvested at 35, 42, 49 and 56 days after induction of flowering in the female plants (Fig. 7). In addition, male vegetative leaf and reproductive tissues (pollen sacs) from the male strain plant were included to complete the transcriptome atlas. Plant samples used in this study include both organs and tissues however, for ease of description the term ‘tissue’ is used to mean both categories throughout.

### Trichome isolation

Trichomes were harvested from the female floral buds using the method described previously^43^ with some modifications. Harvested floral bud tissue (~3–5 cm × 3–5 cm) was placed in a Falcon 50 mL tube filled with 20% of liquid nitrogen. The tube was loosely capped and vortexed for a maximum of 2 min to dislodge the trichomes onto the sides of the tube. The remaining tissue was removed manually from the tube by forceps and the released trichomes were gently resuspended in 1 mL of the lysis buffer from the RNeasy Plant Mini Kit (QIAGEN, Hilden, Germany). The resuspended tissue was filtered through the cell strainer (180 microns) to further purify the trichomes which were immediately processed for extraction of RNA.

### Total RNA extraction and RNA-Seq library preparation

For RNA extraction of trichomes and all other harvested samples of the plant, the RNeasy Plant Mini Kit was used (QIAGEN, Germany) with no modifications from manufacturer’s instructions. Total RNA concentration was measured at the wavelength ratio A260/280 nm using a NanoDrop spectrophotometer (Thermo scientific, USA).

SureSelect Strand-Specific RNA Library Kit (Agilent Technologies, USA) was used to prepare RNA-Seq libraries with no modifications from manufacturer’s instructions. All samples were DNA barcoded with a unique sequence. The generated RNA-Seq libraries were assessed for quality and quantification purposes using D1000 ScreenTape on the TapeStation 2200 platform (Agilent Technologies, USA). RNA-Seq libraries were multiplexed for sequencing to generate a single sample. The multiplexed pooled sample was quantified using the high-sensitivity fluorometric assay (Qubit, Thermo Fisher Scientific, USA) and was sequenced (2 × 150 pair-end) using the HiSeq3000 system (Illumina Inc., USA).

### Sequence data processing and de novo assembly

The sequence data was processed, and *de novo* assembly was generated as described previously^36,37^. Briefly, raw sequence reads were filtered by using Cutadapt v. 1.9^44^ and a custom perl script. Low-quality (reads with >10% bases with Q ≤ 20) and adaptor sequences were omitted from the data. Further filtering involved removal of reads that had ≥3 consecutive Ns with a phred score of ≤20 and any reads that were ≤50 bp. The filtered data was assembled using SOAPdenovo-TRANS^45^ with k-mer size of 51, 69, 73, 75, 91 and 101 to find the optimum k-mer size for the assembly. The resulting contigs and scaffolds from the chosen assembly that had a total length of <240 bp were removed, as these are shorter than a single pair of sequence reads. Transcripts that ranged between 240–500 bp in length and had <10 sequence reads associated with the assembly were also discarded. To further improve the assembly, bubble, fork and complex loci from the SOAPdenovo-TRANS assembly were combined using the CAP3 assembler^46^ (minimum overlap of 50 bp and 95% identity).

### Transcriptome annotation

The generated transcriptome assembly was compared using BLASTX^47^ against the UniRef100 database^48^ with the threshold *E*-value of < 10^−10^. The transcripts were further BLASTN analysed against the previously-generated cannabis transcriptome databases of PK and Finola^18^ and to the CDS of CBDRx genome assembly^19^. Transcripts that displayed a significant match to non-plant databases based on their annotation were removed from further analysis. The assembled transcripts were also assigned gene ontology (GO) terms based on sequence similarity to UniRef100 database. GO terms were retrieved based on UniRef100 identifiers using Retrieve/ID mapping tool of UniProt and their distribution across categories was compared and plotted using WEGO^49,50^.

### Differential gene expression analysis

To analyse differential gene expression, quality trimmed sequence reads from each of the tissue sample were aligned to the generated transcriptome assembly using the BWA-MEM software package^51^ using default parameters. Overall transcriptional activity was determined by normalising read counts using the DESeq method^52^. Principal component analysis (PCA) plot was utilised to visualise and assess the clustering of the data. R Bioconductor package, DESeq2^53^ was used to perform differential gene expression analysis. Benjamini-Hochberg method was used to control the false discovery rate (FDR) by adjusting the p-values^54^. Genes were included for further analysis only if they were defined to be significantly differentially expressed; if the value for Log2 fold changes were either ≥ two-fold or ≤-two-fold with adjusted p-value (Padj) of ≤0.05.

The differential expression analysis was carried out separately for the two variables of tissue type and female floral stage-specific development. To study the differential gene expression across multiple tissue types, the samples were categorised into leaf/stem and root tissues from vegetative plant and reproductive tissues of male and female plants (floral buds with trichomes and trichome tissue). For the study of differential expression of genes during female flower development, differential gene expression analysis was carried out separately for female flowers and trichome tissue harvested at days 35 (Stage 1), 42 (Stage 2), 49 (Stage 3) and 56 (Stage 4) post-induction of flowering. Differentially expressed genes identified between Stage 4 and Stage 1 in flowers and trichome tissue were further categorised functionally using GO Annotation (GOA) classification in CateGOrizer^55^. Results of CateGOrizer were further summarised and visualised in REVIGO^56^ to generate the relevant scatterplots.

### Quantitative PCR analysis

The expression of a randomly selected set of 20 differentially expressed transcripts by the RNA-Seq analysis was re-examined using qRT-PCR analysis. RNA was extracted from vegetative tissues (leaf and root) and reproductive female floral buds (Stage 1 and Stage 4) of the Cannbio-2 strain in three replicates as described above. The primer sequences for the selected transcripts were designed using BatchPrimer3^57^ for qRT-PCR (Supplementary Table S8) with default parameters for the product size of 100 to 130 bp, GC content ranging from 40% to 60% and an optimum annealing temperature between 55 and 60 °C. The F-Box gene was used as an internal reference gene. The qRT-PCR, melting curve analysis and normalisation of the obtained data against the internal control was conducted as detailed previously^36,37^. Average of normalised qRT-PCR data and normalised read count from each of the different samples; leaf, root and reproductive female floral buds (Stage 1 and Stage 4) was used for calculation of Pearson’s correlation coefficient. The correlation between the RNA-Seq and qRT-PCR data was made using R package, ggpubr.

### Expression analysis of genes involved in terpene and cannabinoid synthesis

BLASTN analysis with the threshold *E*-value of <10^−10^ was performed against terpene synthases and the genes involved in terpene synthesis of *C*. *sativa*^24^ to identify the associated transcripts of interest from the current assembly. Additionally, candidate transcripts were identified as tetrahydrocannabinolic acid synthase (THCAS), cannabidiolic acid synthase-like 1 (CBDAS- like 1) and cannabidiolic acid synthase (CBDAS) based on the annotation of similarity results to UniRef100 database. The relative level of expression for these transcripts in each tissue type and across the female reproductive developmental stages was determined by normalised read count analysis. The identified candidate transcripts with normalised read count of over 100 in at least one sample were considered to be expressed significantly and were used to generate relevant heat maps with R Bioconductor packages, gplots and d3heatmap.

## Supplementary information

Supplementary Information

Supplementary Information

## Data Availability

Sequence data has been deposited at DDBJ/EMBL/GenBank under the BioProject ID PRJNA560453.
