# Supplementary material for: Generation of a Comprehensive Transcriptome Atlas and Transcriptome Dynamics in Medicinal Cannabis
Source: Sci Rep. 2019 Nov 12;9:16583. doi: 10.1038/s41598-019-53023-6 (PMC6851104; doi:10.1038/s41598-019-53023-6)
Supplement: Supplementary file 1 — Supplementary Information [file 41598_2019_53023_MOESM1_ESM.pdf]

Generation of a Comprehensive Transcriptome Atlas and Transcriptome Dynamics in Medicinal Cannabis

Shivraj Braich, Rebecca C. Baillie, Larry S. Jewell, German C. Spangenberg, Noel O.I. Cogan

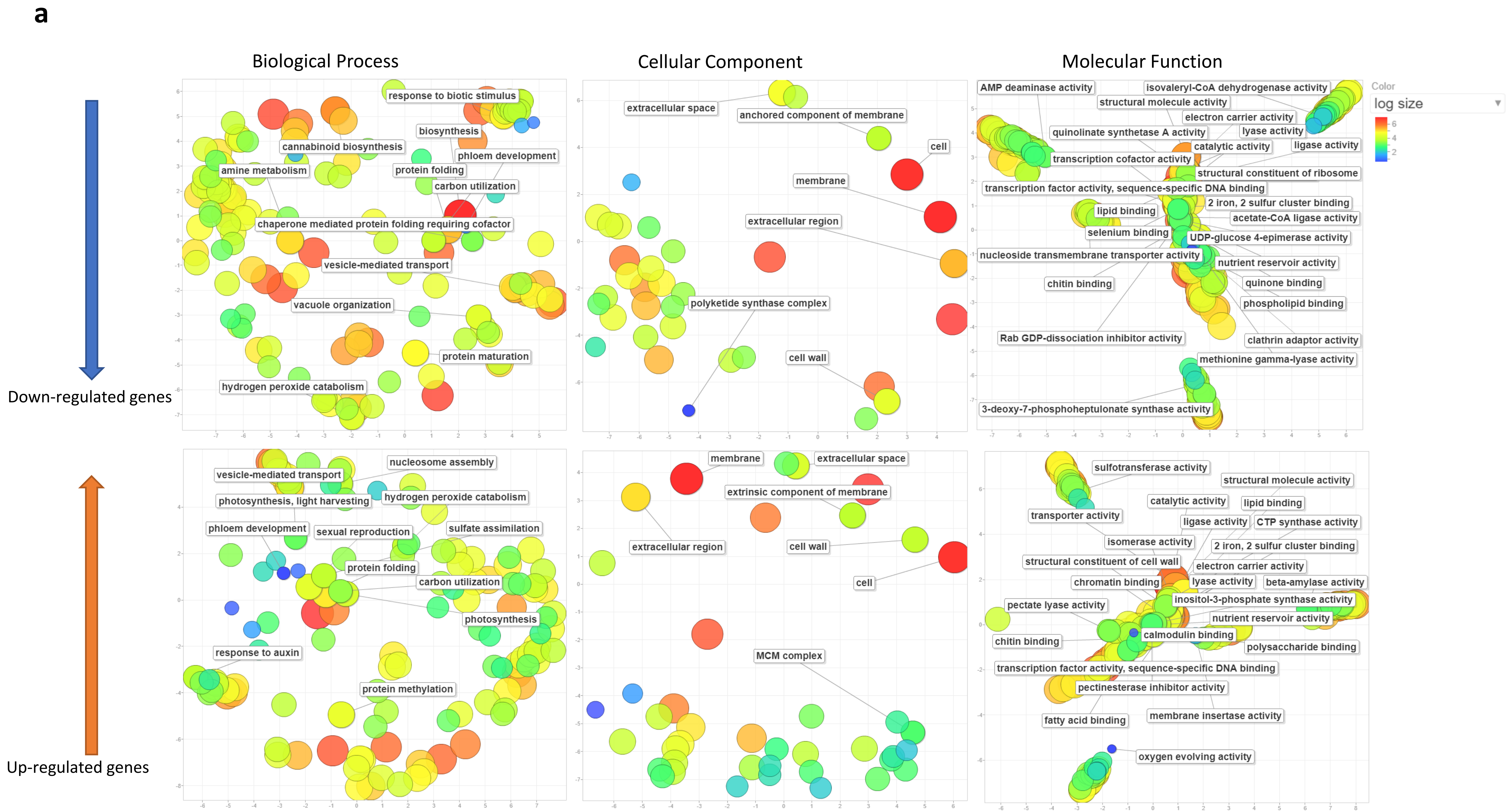

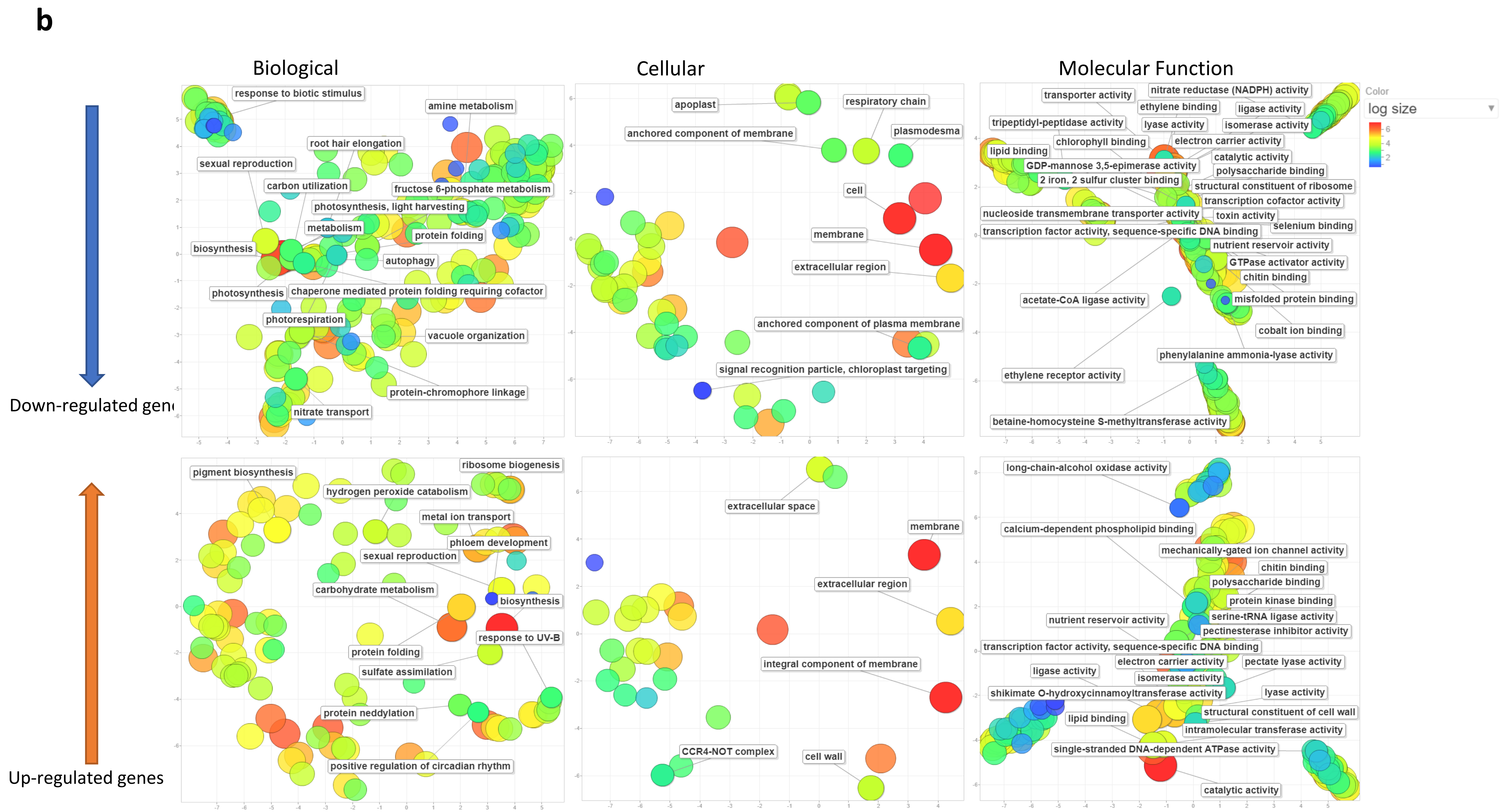

Supplementary Fig. S1. Scatterplot of the summarised GO terms related to biological processes, cellular component and molecular function of differentially expressed genes at Stage 1 when compared to Stage 4 in flowers (a) and trichomes (b). Circle size and color is proportional to the log size of the GO terms, color indicates the uniqueness. Distance between circles is representative of GO terms' semantic similarities. Each of the circles represent a GO term, which depending on the similarity in the terms included in them they will be closer or more distant in the graph.

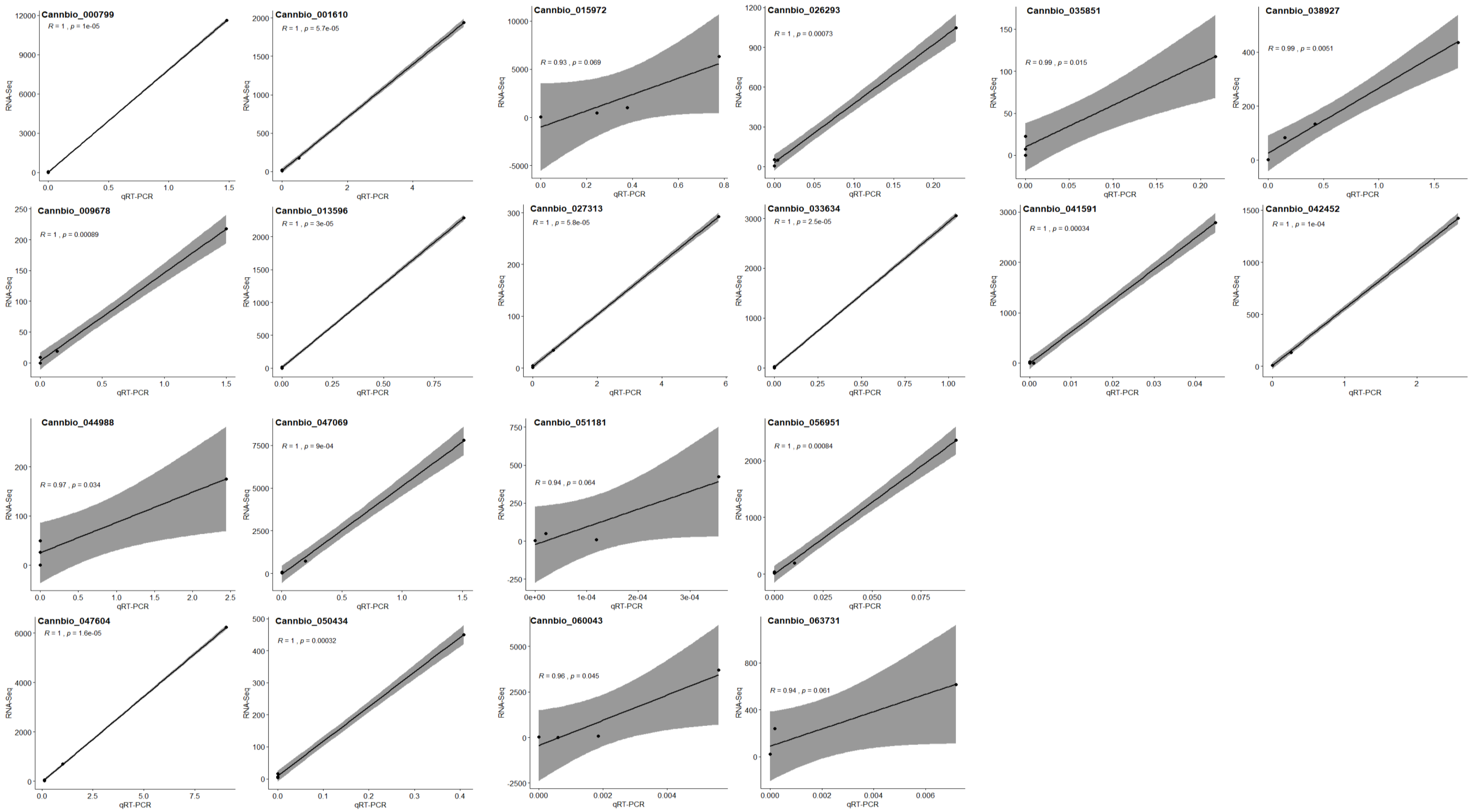

Supplementary Fig. S2. Expression profiles of selected transcripts obtained from qRT-PCR and RNA-Seq from different tissues and the Pearson correlation between expressions measured by qRT-PCR and RNA-Seq analysis.

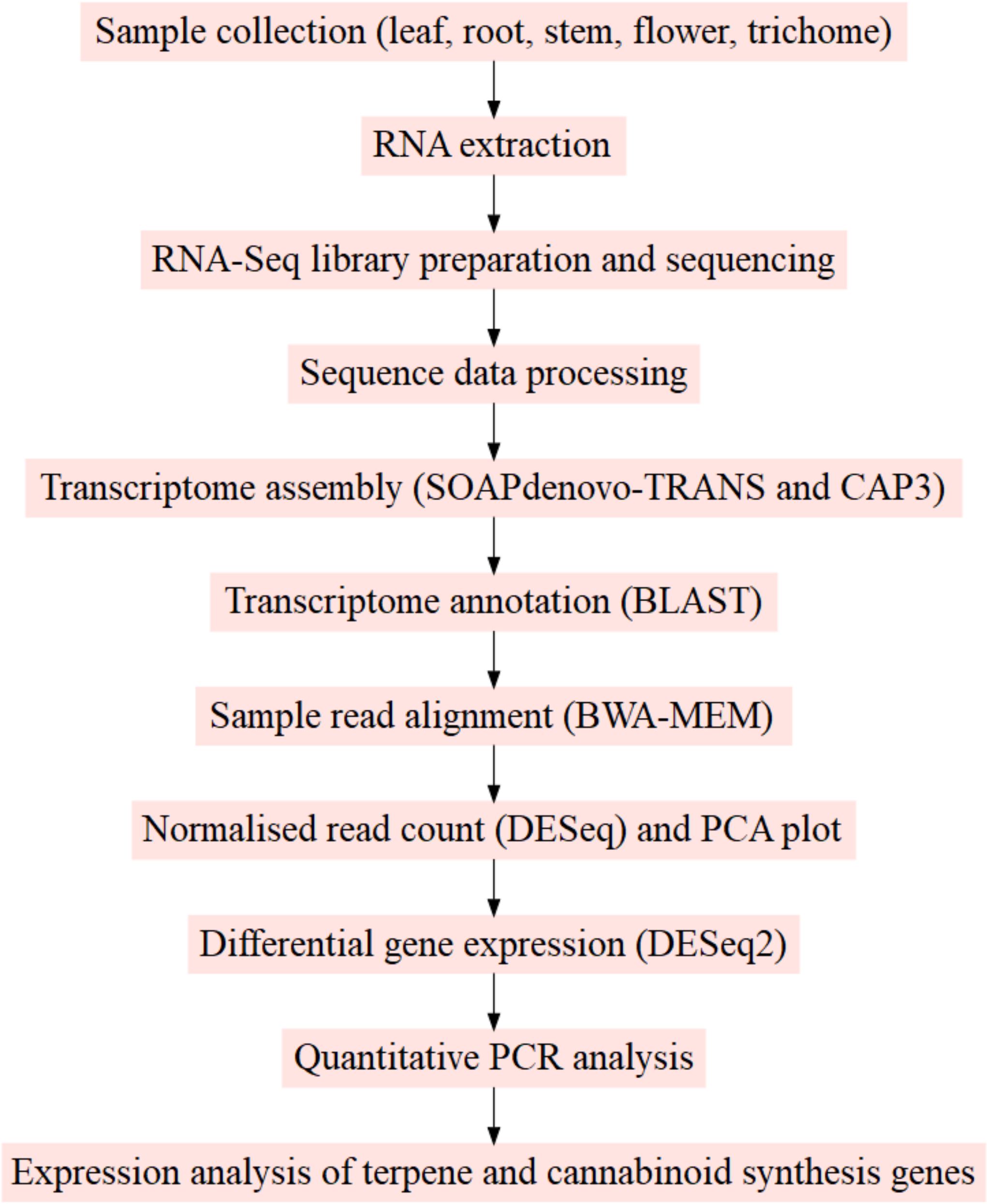

Supplementary Fig. S3. Flow chart of experimental procedure followed in the study for comprehensive transcriptome analysis.
